# Isometric Handgrip Exercise Training Improves Spirometric Parameters and Pulmonary Capacity

**DOI:** 10.3390/pathophysiology28030022

**Published:** 2021-07-20

**Authors:** Ogbutor Udoji Godsday, Nwangwa Eze Kingsley, Nwogueze Bartholomew Chukwuebuka, Chukwuemeka Ephraim, Ezunu Emmanuel, Agbonifo-Chijiokwu Ejime, Igweh John Chukwuka

**Affiliations:** 1 Department of Physiotherapy, Federal Medical Centre Asaba, Asaba PMB 1033, Nigeria; gcclinics@gmail.com (O.U.G.); ephdebest@yahoo.com (C.E.); emmapaulok@gmail.com (E.E.); 2 Department of Human Physiology, Faculty of Basic Medical Sciences, Delta State University, Abraka PMB 1, Nigeria; eknwangwa@delsu.edu.ng (N.E.K.); julicambel@gmail.com (A.-C.E.); jcigweh@delsu.edu.ng (I.J.C.); 3 Department of Human Physiology, College of Health Sciences, Evangel University Akaeze, Akaeze PMB 129, Nigeria

**Keywords:** prehypertension, isometric handgrip exercise, training, maximum voluntary contraction, spirometry

## Abstract

Decline in normal physiological pulmonary function has been attributed to premorbid conditions such as prehypertension. Research evidence suggests that physical activity reduces age-related decline in pulmonary function and improves the efficiency of the lungs in prehypertensive patients. However, there is a scarcity of data evidence relating to isometric exercise and pulmonary function. Furthermore, the interrelationship between the intensity and duration of isometric exercise and pulmonary function in these patients is still uncertain. Therefore, this study was undertaken to investigate the effect of isometric handgrip exercise on pulmonary function capacity in adults with prehypertension. To determine the effectiveness of isometric handgrip exercise on pulmonary function capacity in adults with prehypertension. A quasi experiment using a pre- and post-exercise method was carried out in two out-patients hospital settings. The sample comprised 192 sedentary pre-hypertensive subjects, aged between 30–50 years, that were randomly distributed into three groups of 64 participants each. The subjects performed, for 24 consecutive days, an isometric handgrip exercise at 30% Maximum Voluntary Contraction (M.V.C.). At the end of the 24 days, group one (GP1) discontinued, while group two (GP2) continued the exercise protocol for another 24 consecutive days and group three (GP3) continued with the exercise protocol for another 24 consecutive days but at 50% M.V.C. Determinants of lung function (outcomes) were Forced Expiratory Volume in 1 s (FEV1), Forced Vital Capacity (FVC), FEV1/FVC Ratio and Peak Expiratory Flow Rate (PEFR). The study shows that there was no statistically significant difference in the pre- and post-exercise outcomes for FEV1, FVC, FEV1/FVC Ratio and PEFR after 24 days for group 1. In group 2, there was a statistically significant difference in the FVC [(mean = 0.12 ± 0.12), (*p* = 0.002)], FEV1 [(mean = 0.15 ± 0.17), (*p* = 0.003)] and PEF [(mean = 0.85 ± 0.35), (*p* = 0.001)] after 48 days. In group 3, there was a statistically significant difference (*p* = 0.001) in all the outcomes assessed after 48 days. There was a between groups difference in favour of group 2 compared with group 1 for outcomes of FEV1 [(mean = 0.142 ± 0.68), (*p* = 0.005)] and PEF [(mean = 0.83 ± 0.19), (*p* = 0.0031)]. There was statistically significant difference in favour of group 3 compared to group 2, by increasing the exercise intensity from 30% to 50% M.V.C., for outcomes of FVC [mean change = 0.10 ± 0.052), (*p* = 0.005)], FEV1/FVC [mean change = 3.18 ± 0.75), (*p* = 0.017)] and PEF [(mean change = 0.86 ± 0.35), (*p* = 0.001)] after 48 days. Isometric handgrip exercise (after 48 days at 30% to 50% M.V.C.) improves outcomes of pulmonary function capacity in adults with prehypertension. Meanwhile, duration and/or increase in intensity of the isometric effort significantly contributed to the affects attained.

## 1. Introduction

One in four people experience prehypertension at any point in time globally [1,2]. Research evidence suggests that it causes metabolic and respiratory disturbances that are associated with increased cardiovascular complications, with high morbidity and mortality rates [2,3]. Although it is important to identify the cause of prehypertension and intervene early in the course of the disease, the mechanism of its pathophysiology is unclear [4]. Several studies have revealed that lung function, such as forced expiratory volume in one second (FEV1) and forced vital capacity (FVC), is closely linked to cardiovascular diseases such as hypertension [5,6,7,8]. For example, [8] reported that lung functions—both FVC and FEV1—were reduced in adults with hypertension. The implication is that improved lung functions would have beneficial effects on prehypertensive patients. However, the physiological interactions between the respiratory and cardiovascular systems are not fully known.

Physical exercise is a major component of the total therapeutic regimen in pulmonary rehabilitation [9,10,11] and has been positively correlated with better lung function in all age groups [12,13]. The World Health Organization estimates that, approximately 3.2 million deaths are attributable to insufficient physical exercise each year, making it one of the leading risk factors for cardiovascular disease, such as hypertension, and death throughout the world [14]. Several studies have shown that physical activity may attenuate age-related decline in pulmonary function and should be an integral part of pulmonary rehabilitation to improve lung function [11,15,16]. Respiratory diseases constitute a major socioeconomic and a massive health burden all over the world. It represents an enormous drain on human and financial resources, and also contributes largely to morbidity and mortality at both global and national scales [17]. Sillanpää et al. stated that there is a general age related decline in pulmonary function even in the absence of extrinsic pollutants, which can be caused by several factors related to the lung tissue itself [18]. This age related decline further causes a reduction in exercise capacity and contributes immensely to loss of muscle power and mobility with progressive airflow limitation. Studies have shown that the reduction in muscle power and increased airflow limitation contribute to the loss of muscle mass, decreased functional capacity and eventually loss of independence [15,19].

Research has shown that muscle exercises, such as running, cycling and walking, improve lung function in chronic diseases such as hypertension, and is widely used by both patients and clinicians [11,20]. Despite the awareness of the enormous beneficial effects of physical exercise on lung functions and cardiovascular patients, such as those with prehypertension, compliance to exercise prescriptions is poor. The National Centre for Health Statistics reports that only about 30 percent of adults participate in regular physical exercise [21]. In a study using accelerometers, it was suggested that self-reports of physical activity are over-estimated [22]. This poor compliance has been attributed to the type of physical exercise recommendations. This is because previous exercise recommendations have centred on dynamic exercises, such as jogging, which are not only difficult to quantify but are physically demanding and cumbersome. In some instances, individuals with prehypertension or lung disease may not be able to participate in such exercises. In some instances, individuals, as a result of disabilities or diseases, may not be able to participate in dynamic exercises. Therefore, research should focus on the application and roles of physical activities in the prevention and reversibility of these diseases and effective means of enhancing compliance to recommended physical activities.

One promising regimen, currently being explored, is isometric handgrip exercise training [23]. Handgrip exercises are easy to perform, take less space and are more accessible in various locations such as hospitals, schools, in transit and in the home; therefore, it can lead to increased adherence to treatment. However, available data are scarce with regard to the recommendations for isometric exercise. Wiles et al. stated that, currently, there are no definitive statements, positional declarations nor recommendation guidelines for the use of isometric exercise and that there has been a continued reluctance to promote isometric exercise by committees and organizations that are charged with the responsibility for public health recommendations of physical activity [24]. A good number of scientific studies clearly demonstrate that isometric exercise is efficacious in the attenuation of resting blood pressure in both normotensive and hypertensive subjects [24,25,26,27,28]. Meta-analysis by different authors reports that isometric exercise produced greater reductions in systolic and diastolic blood pressure compared to dynamic exercise training [29,30]. Moreover, physical exercise has been shown to be a non-pharmacological prophylactic regimen as it is capable of protecting against decline in pulmonary functions due to aging and enhances the physiological responses of the lungs [10,11,15].

Pulmonary functions are usually determined by the compliance of the thoracic cavity, respiratory muscle strength, airway resistance and elastic recoil of the lungs. Exploring a physical exercise mode which could be beneficial and promote and preserve efficient lung function is an essential preventive strategy in this busy age. It is therefore imperative to examine a low dose isometric hand grip exercise effect in the pulmonary system. Additionally, isometric exercise has been shown to be comparable to dynamic exercise with regards to the development of muscle bulk, intramuscular and peak rate of tension and muscle strength and endurance [24]. Furthermore, isometric exercise has the advantages of ease of access and use and especially enables those with co-morbidities that may restrict movement to participate. Prominent aspects of isometric handgrip exercise include its ease and speeds of performance, which can be achieved in any location with less concentration. These attributes may enhance compliance thereby increasing the probability of positive clinical outcomes. However, the evidence supporting the effectiveness of isometric handgrip exercise training on pulmonary functions, such as FEV1, FVC, FEV1/FVC Ratio and Peak Expiratory Flow Rate (PEFR), in adults with prehypertension is scarce. Since lung functions such as FVC and FEV1 are related to cardiovascular diseases such as hypertension, and isometric exercise improves hypertension, it is important to investigate the effectiveness of isometric handgrip exercise on pulmonary functions in prehypertensive individuals. Therefore, this study is aimed at investigating the effectiveness of isometric handgrip exercise on spirometric parameters in adults with prehypertension. The research question for this study was: Is isometric handgrip exercise effective at improving spirometric parameters such as FEV1, FVC, FEV1/FVC and PEFR in adults with prehypertension?

## 2. Materials and Methods

### 2.1. Experimental Design

All the subjects were diagnosed and referred by the physician with a blood pressure level classified as prehypertension based on the classification of the Joint National Committee on Prevention, Detection, Evaluation, and Treatment of High Blood Pressure. This represents a systolic blood pressure range of 120–139 mmHg and a diastolic blood pressure range of 80–89 mmHg. Only subjects who were physically and clinically in good health, devoid of any pulmonary, cardiovascular, haematological and clinical abnormalities, were recruited into the study. Subjects were not on medication and all the participants were untrained, which was defined by a score of three or less using the Rapid Assessment of Physical Activity survey [31]. The subjects had a mean age of 40 ± 10 years. This is because pulmonary system degenerative diseases and prehypertension have been found to have an increased risk in individuals 40 years and above. Subjects were excluded from the study if they suffer from debilitating arthritis, carpel tunnel, peripheral neuropathy, an aneurysm, or mitral valve complications. The subjects were properly briefed and written informed consent was obtained. This study jointly received institutional ethical approval from the Federal Medical Centre, Asaba, Delta State (FMC/ASB/A81.VOL. XII/101) and the Faculty of Basic Medical Sciences, Delta State University, Abraka, Delta State (REC/FBMS/DELSU/18/16/103), and conformed to the 1964 Helsinki declaration and its later amendments or comparable ethical standards.

### 2.2. Subjects

The general study design consisted of an isometric handgrip exercise trial. One hundred and ninety two prehypertensives (n = 192, males = 105 and females = 87, age, 39.04 ± 6.4 years; body mass index, 25.45 ± 2.72 kg/m^2^) were recruited for the study. Inclusion in the study was subject to a normal medical examination, determined by a consultant physician.

### 2.3. Experimental Methods

The procedure for this study involved the study of the medical case notes and assessment of the subjects. Information about diseases, medication and smoking were self-reported using both a questionnaire and verbally during a medical examination. A screening session was conducted to assess the baseline parameters and blood pressure of the subjects and spirometric data were collected. The sample population was randomly allocated into one of the three groups. The subjects were asked to pick from a ballot box concealed papers marked EG1, EG2 or EG3 to determine to which group the subjects should belong. A detailed procedure of the exercise was then given to the subjects before commencement of the exercise training. On arrival at the clinic on the first day, subjects were made to observe a 15 min seated rest after which their spirometric data were collected for baseline levels of the forced vital capacity, forced expiratory volume in 1 s, Tiffeneau-Pinelli Index and peak expiratory flow respectively (FVC, FEV_1_, FEV_1_/FVC and PEF respectively). A detailed explanation and a demonstration of the exercise protocol were given to the subjects and they were asked to report to the Physiotherapy clinic at 4.00 pM for the exercise daily. The training session for each day took place between the hours of 4.00 pM and 8.00 pM daily.

The subjects on arrival at the clinic were made to observe a 15 min seated rest after which they were asked to squeeze the dynamometer with their dominant hand twice, for a maximum of 2 s with a five minute rest in between, so as to determine their respective maximum voluntary contraction (M.V.C.) for each session. The mean of the two readings was taken as the M.V.C. for each subject for that session. Subjects were thereafter instructed to squeeze and sustain the dynamometer for 2 min at 30% M.V.C. The dynamometer pointer which read the scale provided visual feedback to the subjects for the maintenance of the 30% M.V.C. This procedure was repeated twice for each training session with a 5 min rest in between. The position adopted by the subjects throughout the exercise training was sitting with upper limbs supported on a table. The exercise protocol was performed for 24 consecutive days. Group one (G1) discontinued with the exercise protocol after 24 days while group two (G2) continued for another 24 consecutive days at 30% M.V.C. On the other hand, group three (G3) continued with the exercise protocol for another 24 consecutive days but at 50% M.V.C. Blood samples were collected again on the 49th day for assessment of the resting data of the forced vital capacity (FVC), forced expiratory volume in 1 s (FEV_1_), Tiffeneau-Pinelli Index (FEV_1_/FVC ratio) and peak expiratory flow (PEF), respectively.

The spirometric data were collected following the recommended guidelines. The equipment was properly calibrated following the manufacturer’s instructions. Spirometry was performed according to the American Thoracic Society, [32] recommendations, which require that no physical exercise should be done in the last 3 h. The position adopted by the subjects was upright sitting with the knees and hip flexed at 90° and wearing a nose clip. The participants were asked to blow into the mouthpiece of the spirometer forcefully and quickly and to continue blowing until all of the air was expelled from the lungs. A pneumotachograph-type spirometer (Spirolab 111alar, MIR009 by Medical International Research, Viadel Maggiolino 1250015, Rome, Italy) was used, which has demonstrated a volume accuracy of ±3% with no significant nonlinearity. Measurements were repeated until grade A was obtained and the highest value was used in the analysis. Pulmonary function was assessed based on Forced Expiratory Volume in first second (FEV1), Forced Vital Capacity (FVC), Tiffeneau index (FEV1/FVC) and Peak Expiratory Flow (PEF).

### 2.4. Inclusion and Exclusion Criteria

The subjects had a mean age of 40 ± 10 years. This is because pulmonary system degenerative diseases and prehypertension have been found to have an increased risk in individuals 40 years and above. Subjects were excluded from the study if they suffered from debilitating arthritis, carpel tunnel, peripheral neuropathy, an aneurysm, or mitral valve complications. A screening session was conducted to assess the baseline parameters and blood pressure of the subjects and spirometric data were collected. The sample population was randomly allocated to one of the three groups. The subjects were asked to pick from a ballot box concealed papers marked G1, G2 or G3 to determine to which group the subjects should belong. A detailed procedure of the exercise was then given to the subjects before commencement of the exercise training.

### 2.5. Data Collection

The equipment was properly calibrated following the manufacturer’s instructions. Spirometry was performed according to American Thoracic Society, [32] recommendations, which require that no physical exercise should be done in the last 3 h. The position adopted by the subjects was upright sitting with the knees and hip flexed at 90° and wearing a nose clip. The participants were asked to blow into the mouthpiece of the spirometer forcefully and quickly and to continue blowing until all of the air was expelled from the lungs. A pneumotachograph-type spirometer (Spirolab 111alar, MIR009 by Medical International Research, ViadelMaggiolino 1250015, Rome, Italy) was used, which has demonstrated a volume accuracy of ±3% with no significant nonlinearity. Measurements were repeated until grade A was obtained and the highest value was used in the analysis. Pulmonary function was assessed based on Forced Expiratory Volume in first-second (FEV1), Forced Vital Capacity (FVC), Tiffeneau index (FEV1/FVC) and Peak Expiratory Flow (PEF).

### 2.6. Data Analysis

All analyses were conducted using SPSS version 21 (SPSS Inc., Chicago, IL, USA) and the significance level was determined as *p* < 0.05. The collected data were descriptively and inferentially analysed. The descriptive statistics employed in this study were the mean and standard deviation representation. The inferential statistics used in the analysis of the data included a one tailed student’s *t*-Test to determine the intra-groups differences in the initial and final values of the parameters, of the three groups. One way analysis of variance was thereafter used to compare the means of the three groups to determine their level of significance. Furthermore, the one tailed student’s *t*-Test was used to determine the intra-groups differences in the initial and final resting values of the parameters of group one and group two and group two and group three to determine the effect of duration and intensity of the isometric effort respectively.

## 3. Results

The results of this work provide evidence on the effect of isometric handgrip exercise therapy on pulmonary function.

Subjects Characteristics

Table 1 shows the descriptive demographic data of the group participants; a total of one hundred and ninety two (192) subjects with a mean age of 39.04 ± 6.4 years and body mass index of 25.4 ± 2.7 kg/m^2^ participated in and completed the programme.

Table 2 above shows the pre and post exercise mean values of the spirometric indices across the three exercise groups. In EG1, there was a mean reduction of 0.02 ± 0.11 L and 0.02 ± 0.16 L/min in the forced vital capacity and peak expiratory flow rate, respectively, and a mean increase of 0.01 ± 0.7 L/s and 0.21 ± 1.57% of forced expiratory volume in one second (1 s) and FEV_1_/FVC ratio, respectively. On the other hand, EG2 participants’ showed an increase of 0.12 ± 0.12 L, 0.15 ± 0.17 L/s, 0.28 ± 3.9% and 0.85 ± 0.4 L/min of forced vital capacity, forced expiratory volume in 1 s and FEV_1_/FVC ratio and in peak expiratory flow rate, respectively. In EG3, the participants had an increase in all the variables of 0.22 ± 0.07 L, 0.23 ± 0.2 L/s, 3.46 ± 3.15% and 1.71 ± 0.7 L/min of forced vital capacity, forced expiratory volume in one second (1 s), FEV_1_/FVC ratio and peak expiratory flow rate, respectively.

Table 3 shows an independent t-test of significance for the pulmonary parameters of the three exercise groups at the end of 48 days. In EG1, the result shows no significant change in all the variables analysed. This shows that cessation of the exercise protocol after 24 days did not produce any statistical change in the values of the spirometric indices analysed. It is possible that the exercise duration was inadequate to produce a statistical change in the parameters assessed or that the changes recorded at the end of 24 days had reversed to the pre-training level. In EG2, the results show a statistically significant difference in the FVC, FEV_1_ and PEF. This means that continuation of the exercise protocol for 48 days produced a significant increase in the FVC, FEV_1_ and PEF but not in the FEV_1_/FVC ratio. The lack of significance in the FEV_1_/FVC ratio may be due to the concomitant increase in the values of FVC and FEV_1_. On the other hand, EG3 participants’ results show a statistically significant difference in all the parameters assessed. This means that an increase of the exercise dosage produced a significant increase in the pulmonary indices at the end of 48 consecutive days.

Table 4 shows the comparative changes in the mean values of the spirometry parameters between EG1 (Cessation) and EG2 (Continuation). The table shows an increase in all the variables with average values of 0.091 ± 0.01 L, 0.142 ± 0.68 L/s, 0.075 ± 2.33% and 0.83 ± 019 L/min in the FVC, FEV_1_, FEV_1_/FVC and PEF, respectively, in favour of EG2. This means that continuation of the exercise protocol produced more effects in these variables compared to the cessation of the exercise protocols after 24 days. The independent t-test of significance for EG1 and EG2 shows a significant difference in the values ofFEV_1_ and PEF but no significant difference was observed for FVC and FEV_1_/FVC. This means that continuation of the exercise protocol as seen in group two produced a significant increase in the FEV_1_ and PEF compared to the cessation of the exercise protocols after 24 days but did not produce a significant increase in FVC and FEV1/FVC.

Table 5 shows the comparative effects of continuation (EG2) and increase in intensity (EG3). The results show an increase in all the variables with average values of 0.10 ± 0.052 L, 0.08 ± 0.01 L/s, 3.18 ± 0.75% and 0.86 ± 0.35 L/min in FVC, FEV_1_, FEV_1_/FVC and PEF, respectively, in favour of EG3. This means that increasing the exercise intensity resulted in an increase in these values as compared to continuing the exercise protocol. These values were statistically significant except for the FEV_1_. This means that increasing the exercise intensity from 30% to 50% M.V.C. as seen in group 3 produced a more significant increase in the values of the parameters analysed compared to the continuation of the exercise protocols, except in the value of FEV_1_.

Table 6 shows a one way analysis of variance (ANOVA) on the effects of the three exercise groups. The table shows a significance difference in all the variables analysed. This means that a significant difference exists inthe effects noted in the three exercise protocols on all the parameters of the pulmonary system analysed.

## 4. Discussion

This study set out to investigate the effectiveness of isometric handgrip exercise on pulmonary function capacity in adults with prehypertension. The study findings demonstrated that, in normal out-patient settings, isometric handgrip exercise improves the outcomes of pulmonary function capacity (FVC, FEV1, FEV1/FVC and PEF) in adults with prehypertension after 48 days at 30% M.V.C. Furthermore, pulmonary functions were found to be improved simultaneously with increased exercise intensity and duration at 50% M.V.C. A total of 192 participants with a mean age, 39.04 ± 6.4 years and BMI, 25.4 ± 2.7 kg/m^2^ participated and completed the study with no loss to follow-up.

### 4.1. Within Group Difference

The study shows that there were no statistically significant differences in the pre- and post-exercise outcomes for FEV1, FVC, FEV1/FVC Ratio and PEFR after 24 days for group 1. The plausible explanation of this is that 24 consecutive days of isometric handgrip exercise at 30% M.V.C. was not adequate for eliciting a statistical change in the outcomes measured at the end of 24 days. Participants’ lung function might have reversed towards pre-training level at the end of 24 days due to exercise cessation. However, in group 2, there was a statistically significant difference in the FVC [(mean = 0.12 ± 0.12), (*p* = 0.002)], FEV1 [(mean = 0.15 ± 0.17), (*p* = 0.003)] and PEF [(mean = 0.85 ± 0.35), (*p* = 0.001)] after 48 days. In group 3, there was a statistically significant difference (*p* = 0.001) in all the outcomes assessed after 48 days. The implication of these outcomes is that the continuation of the exercise protocol for a longer duration produced better lung function in patients with prehypertension. However, future research might be necessary to measure the effectiveness of isometric handgrip on pulmonary functions in prehypertensive adults at the 25th day and at the 49th day to ensure longer time effects.

### 4.2. Between Group Difference

There was a between group difference in favour of group 2 compared with group 1 for outcomes of FEV1 [(mean = 0.142 ± 0.68), (*p* = 0.005)] and PEF [(mean = 0.83 ± 0.19), (*p* = 0.0031)]. There was a statistically significant difference in favour of group 3 compared to group 2, by increasing the exercise intensity from 30% to 50% M.V.C. in group 3, for outcomes of FVC [mean change = 0.10 ± 0.052), (*p* = 0.005)], FEV1/FVC [mean change = 3.18 ± 0.75), (*p* = 0.017)] and PEF [(mean change = 0.868 ± 0.35), (*p* = 0.001)] after 48 days. These findings show, therefore, that increased exercise intensity and duration contributes directly to improvements in pulmonary function in prehypertensive adults. The findings of the study are consistent with previous research [10,11,33,34,35,36,37], which have demonstrated significant improvements in several lung functions using physical training. For example, a previous study reported beneficial effects of physical training of 9 months duration on pulmonary function tests in a cohort of Border Security Force (BSF) trainees compared to that in 100 healthy medical student matched controls [10]. Similar findings have been reported by others investigating the effectiveness of physical exercise with obese participants [33]. A previous study found that physical activities for 90 min every day for one year substantially improved FVC, FEV1, PEF and FEV1% in the physically active group compared with their sedentary counterparts [34]. The work of [34] compares favourably with [35], who found that the pulmonary functions of athletes who performed regular and long-term exercises were better compared with sedentary individuals who were not interested in any sporting activity.

However, this study has some shortcomings—the authors did not clearly define the type and intensity of the exercise of the intervention group. Further still, there was no control over type of exercise, the frequency and the exact timing for each exercise. The implication of these limitations is that the results of this study are potentially questionable. By contrast, there was also no observation of possible decline in lung function after cessation of physical activity in relation to this present study where there was no significant improvement in post exercise lung outcomes after 24 days for group 1 [35]. Another study investigated the effect of training intensity and sporting type on pulmonary function parameters in a cohort of 35 soccer and 35 futsal athletes [36]. The authors found that the soccer athletes’ group had a significantly higher value of FVC, FEV1, PEFR and FEV1/FVC ratio compared with the futsal athletes’ group. They concluded that regular strong and long-duration exercise, as well as the types of training, produces a positive effect on lung function by increasing pulmonary capacity and function. Their work was able to prove that higher intensity and longer duration exercise could produce more effects than lower intensity exercise, considering the requirements of the two groups. Although the work of these previous authors [10,33,34,36] support the use of physical exercise to improve lung function, they had performance bias (differences in the care received by the intervention and control groups in a study) because they did not employ a standardized quantifiable exercise modality.

The implication is that the findings of these studies should be interpreted with caution. However, Ref. [38] studied the acute effects of ongoing isometric handgrip exercises on the in spiratory (IRV) and expiratory reserve volumes (ERV). The other outcomes were FEV1, PEFR and FEV1/FVC and were measured both at baseline and during isometric handgrip exercise. They found that FVC significantly reduced during the exercise but no significant change was observed in the other parameters. They concluded that the reserve capacity of the lung does not change significantly during isometric exercise. The authors also pointed out the limitation of the use of hand grip exercise—only the upper and pectoral muscles were contracting during the procedure. The implication is that the effect of the hand grip exercise on the respiratory parameters may not be as pronounced compared to those exercises involving many muscle groups, for example, in isotonic exercises such as walking and jogging [39,40]. Other studies further explained that the observed improvement in pulmonary function, as indicated by the positive relationships between physical exercise and spirometric outcomes, may be explained partly by muscle strength, rather than by the total activity such as handgrip strength, which has been associated with improved spirometry in a cohort of healthy adolescents [11,41,42,43,44].

## 5. Conclusions

In conclusion, this study has shown that isometric handgrip exercise should be incorporated into daily routine pulmonary rehabilitation in a quantifiable manner because there is evidence that it improves pulmonary function, which means it can minimize the rate of morbidity and mortality associated with age related pulmonary decline. The improvement in the lung function parameters observed in groups 2 and 3 implies that long term exercise duration and increased intensity are beneficial in prehypertensive adults. However, the results of this study should be interpreted with caution because participants were adequately randomized to intervention and control group. However, a non-randomized study can serve as a basis and motivation for a future randomized control trial (RCT) [45]. Therefore, further research may be necessary to investigate measurements of pulmonary functions at the 25th day and at the 49th day to establish longer term effects using RCT.

## Figures and Tables

**Table 1:** Descriptive demographic data of the group participants.

Parameters	N	Mean	Std. Deviation
Age	192	39.04	6.441
Height	192	1.7000	0.11299
Weight (kg)	192	73.3750	9.00975
BMI (kg/m^2^)	192	25.4487	2.72359

**Table 2:** Pre and post exercise mean values of the spirometric parameters at the end of 48 days.

Parameter		FVC (L)	FEV1 (L/s)	FEV1/FVC (%)	PEF (L/min)
Exercise	Pre	3.31 ± 0.49	3.07 ± 0.55	78.37 ± 7.13	7.04 ± 1.38
Group 1	Post	3.29 ± 0.45	3.08 ± 0.55	78.58 ± 6.8	7.02 ± 1.40
	Diff.	−0.02 ± 0.11	0.01 ± 0.7	0.21 ± 1.57	−0.02 ± 0.2
Exercise	Pre	3.35 ± 0.4	3.10 ± 0.5	79.69 ± 4.22	6.55 ± 1.32
Group 2	Post	3.47 ± 0.4	3.26 ± 0.45	79.97 ± 4.50	7.40 ± 1.44
	Diff.	0.12 ± 0.12	0.15 ± 0.17	0.28 ± 3.9	0.85 ± 0.4
Exercise	Pre	3.30 ± 0.51	2.99 ± 0.39	80.03 ± 3.89	6.67 ± 1.61
Group 3	Post	3.52 ± 0.48	3.23 ± 0.45	83.5 ± 3.21	8.38 ± 1.11
	Diff.	0.22 ± 0.07	0.23 ± 0.2	3.46 ± 3.15	1.71 ± 0.7

Values are expressed as mean ± Standard Deviation (S.D.), n = 192.

**Table 3:** Test of significance of the pre and post exercise mean values of the spirometric parameters.

Groups		Mean Diff.	Sig. (2-Tailed)	Remark
Exercise Group 1	FVC (L)	0.019 ± 0.11	0.626	Insignificant
	FEV_1_ (L/s)	0.0081 ± 0.7	0.662	Insignificant
	FEV_1_/FVC (%)	0.205 ± 1.57	0.61	Insignificant
	PEF (L/min)	0.023 ± 0.16	0.576	Insignificant
Exercise Group 2	FVC (L)	0.12 ± 0.12	0.002 *	Significant
	FEV_1_ (L/s)	0.15 ± 0.17	0.003 *	Significant
	FEV_1_/FVC (%)	0.28 ± 3.9	0.78	Insignificant
	PEF (L/min)	0.85 ± 0.35	<0.001 *	Significant
Exercise Group 3	FVC (L)	0.22 ± 0.07	<0.001 *	Significant
	FEV_1_ (L/s)	0.23 ± 0.18	<0.001 *	Significant
	FEV_1_/FVC (%)	3.46 ± 3.15	0.001 *	Significant

Values are expressed as mean ± Standard Deviation (S.D.), n = 192. * *p* < 0.05.

**Table 4:** The comparative pulmonary effects of cessation and continuation (duration) of the exercise protocol at the end of 48 days.

Parameters	Exercise Group 1	Exercise Group 2	Diff.	Df	Sig. (2-Tailed)	Remark
FVC (L)	0.019 ± 0.11	0.12 ± 0.12	0.091 ± 0.01	127	0.424	Insignificant
FEV_1_ (L/s)	0.0081 ± 0.7	0.15 ± 0.17	0.142 ± 0.68	127	0.005 *	Significant
FEV_1_/FVC (%)	0.205 ± 1.57	0.28 ± 3.9	0.075 ± 2.33	127	0.944	Insignificant
PEF (L/min)	0.023 ± 0.16	0.85 ± 0.35	0.83 ± 0.19	127	<0.001 *	Significant

Values are expressed as mean ± Standard Deviation (S.D.), n = 128. * *p* < 0.05.

**Table 5:** The comparative pulmonary effects of continuation and increase in intensity of the exercise protocol at the end of 48 days.

Parameters	Exercise Group 2	Exercise Group 3	Diff.	Df	Sig. (2-Tailed)	Remark
FVC (L)	0.12 ± 0.12	0.22 ± 0.068	0.10 ± 0.052	127	0.005 *	Significant
FEV_1_ (L/s)	0.15 ± 0.17	0.23 ± 0.18	0.08 ± 0.01	127	0.199	Insignificant
FEV_1_/FVC (%)	0.28 ± 3.9	3.46 ± 3.15	3.18 ± 0.75	127	0.017 *	Significant
PEF (L/min)	0.85 ± 0.35	1.71 ± 0.70	0.86 ± 0.35	127	<0.001 *	Significant

Values are expressed as mean ± Standard Deviation (S.D.), n = 128. * *p* < 0.05.

**Table 6:** Analysis of variance on the effects of the three exercise groups on the spirometric parameters.

Parameters		Sum of Squares	Df	Mean Square	F	Sig.
FVC (L)	Between Groups	0.132	2	0.066	9.170	<0.001 *
	Within Groups	0.323	189	0.007		
	Total	0.455	191			
FEV_1_ (L/s)	Between Groups	0.412	2	0.206	14.840	<0.001 *
	Within Groups	0.625	189	0.014		
	Total	1.037	191			
FEV_1_ FVC (%)	Between Groups	72.485	2	36.242	7.973	0.001 *
	Within Groups	204.542	189	4.545		
	Total	277.026	191			
PEF (L/min)	Between Groups	20.286	2	10.143	48.164	<0.001 *
	Within Groups	9.477	189	0.211		
	Total	29.763	191			

Values are expressed as mean ± Standard Deviation (S.D.), n = 192. * *p* < 0.05.

## Data Availability

Not applicable.
